# Contaminant Exposure and Transport from Three Potential Reuse Waters within a Single Watershed

**DOI:** 10.1021/acs.est.2c07372

**Published:** 2023-01-10

**Authors:** Jason R. Masoner, Dana W. Kolpin, Isabelle M. Cozzarelli, Paul M. Bradley, Brian B. Arnall, Kenneth J. Forshay, James L. Gray, Justin F. Groves, Michelle L. Hladik, Laura E. Hubbard, Luke R. Iwanowicz, Jeanne B. Jaeschke, Rachael F. Lane, Richard Blaine McCleskey, Bridgette F. Polite, David A. Roth, Michael B. Pettijohn, Michaelah C. Wilson

**Affiliations:** † U.S. Geological Survey, Oklahoma City, Oklahoma 73116, United States; ‡ U.S. Geological Survey, Iowa City, Iowa 52240, United States; § U.S. Geological Survey, Reston, Virginia 20192, United States; ∥ U.S. Geological Survey, Columbia, South Carolina 29210, United States; ⊥ Oklahoma State University, Stillwater, Oklahoma 74078, United States; # U.S. Environmental Protection Agency, Ada, Oklahoma 74820, United States; ∇ U.S. Geological Survey, Lakewood, Colorado 80225, United States; ○ U.S. Geological Survey, Sacramento, California 95819, United States; ◆ U.S. Geological Survey, Madison, Wisconsin 53711, United States; ¶ U.S. Geological Survey, Kearneysville, West Virginia, 25430, United States; †† U.S. Geological Survey, Lawrence, Kansas 66049, United States; ‡‡ U.S. Geological Survey, Boulder, Colorado 80303, United States

**Keywords:** wastewater effluent, urban stormwater, agricultural runoff, emerging contaminants, reuse, water reclamation, pharmaceuticals, PFAS, pesticides, polycyclic aromatic hydrocarbons

## Abstract

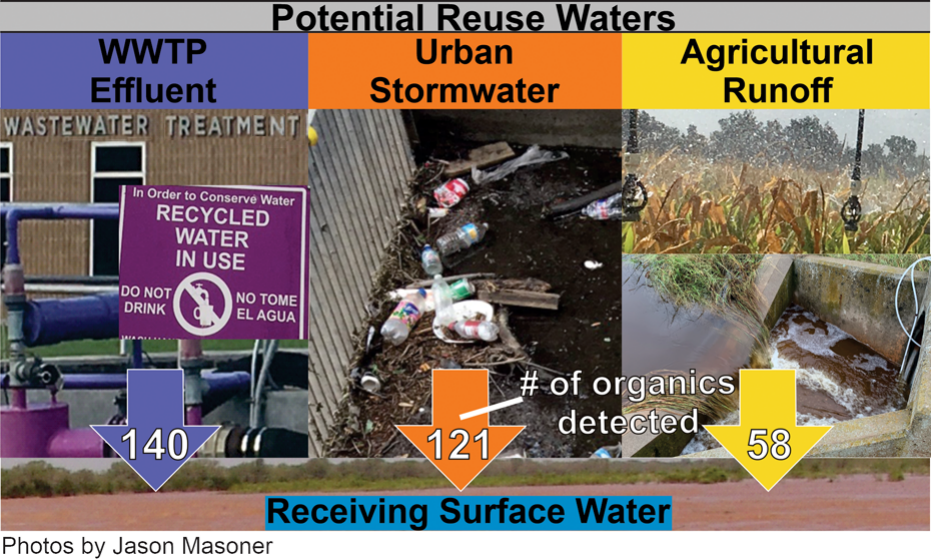

Global demand for safe and sustainable water supplies necessitates a better understanding of contaminant exposures in potential reuse waters. In this study, we compared exposures and load contributions to surface water from the discharge of three reuse waters (wastewater effluent, urban stormwater, and agricultural runoff). Results document substantial and varying organic-chemical contribution to surface water from effluent discharges (e.g., disinfection byproducts [DBP], prescription pharmaceuticals, industrial/household chemicals), urban stormwater (e.g., polycyclic aromatic hydrocarbons, pesticides, nonprescription pharmaceuticals), and agricultural runoff (e.g., pesticides). Excluding DBPs, episodic storm-event organic concentrations and loads from urban stormwater were comparable to and often exceeded those of daily wastewater-effluent discharges. We also assessed if wastewater-effluent irrigation to corn resulted in measurable effects on organic-chemical concentrations in rain-induced agricultural runoff and harvested feedstock. Overall, the target-organic load of 491 g from wastewater-effluent irrigation to the study corn field during the 2019 growing season did not produce substantial dissolved organic-contaminant contributions in subsequent rain-induced runoff events. Out of the 140 detected organics in source wastewater-effluent irrigation, only imidacloprid and estrone had concentrations that resulted in observable differences between rain-induced agricultural runoff from the effluent-irrigated and nonirrigated corn fields. Analyses of pharmaceuticals and per-/polyfluoroalkyl substances in at-harvest corn-plant samples detected two prescription antibiotics, norfloxacin and ciprofloxacin, at concentrations of 36 and 70 ng/g, respectively, in effluent-irrigated corn-plant samples; no contaminants were detected in noneffluent irrigated corn-plant samples.

## Abstract 2

Water management agencies worldwide are increasing water reuse to augment freshwater supplies. This study reports on the mobilization and transport of reuse-derived contaminants to surface from three reuse waters.

## Introduction

Municipalities and water management agencies worldwide are increasingly using municipal wastewater treatment plant (WWTP) effluent,^1,2^ urban stormwater,^3,4^ and agricultural runoff^5,6^ for various water reclamation (water reuse) purposes to meet increasing water supply demands.^7^ Previous studies have documented that effluent,^8−10^ stormwater,^11−13^ and agricultural runoff^14−16^ are sources of complex organic-chemical mixtures that include designed bioactive chemicals (e.g., pharmaceuticals, pesticides), known carcinogens (e.g., polycyclic aromatic hydrocarbons), and endocrine-disrupting/hormonally active chemicals (e.g., biogenic hormones, bisphenol A). These and other contaminants of emerging concern (CECs) are transported and released to the environment by continuous discharge or episodic rain-induced runoff events from urban and agricultural landscapes, resulting in degraded water and soil quality. Endocrine-disrupting chemicals (EDCs) have the potential to induce a hormone-receptor response that can cause adverse health effects in animals and humans.^17^ Estrogens and other EDCs have been found in WWTP effluent,^18^ stormwater,^19^ and agricultural runoff.^20^ WWTP effluent can be used for irrigation in agriculture as a beneficial source of water to increase crop yields, but there is evidence for potential human and animal exposures from food consumption of plants that have taken up WWTP-derived contaminants^21−24^ including per-/polyfluoroalkyl substances (PFAS).^25^ Although previous studies have documented mobilization of such contaminants from fields treated with municipal biosolids,^26,27^ source effluent irrigation,^15^ and surface runoff from excess effluent irrigation,^16^ there are few data that include a broad suite of organic chemicals in rain-induced agricultural runoff from effluent-irrigated fields. Furthermore, there is growing environmental health concern about effects to terrestrial organisms, alterations to natural soil function, and antimicrobial resistance from the release of CECs throughout the environment and food web.^28^ Antimicrobial resistance weakens the effectiveness of antibiotics to fight infections and is recognized as a pervasive global health threat.^29,30^ A global assessment released in May 2016 estimated that antibiotic-resistant bacteria could be responsible for 700,000 deaths annually, and the annual death toll would increase to 10 million deaths per year by 2050.^31^

The myriad chemicals present in effluent,^32^ stormwater,^13^ and agricultural runoff,^33,34^ commonly used as reuse waters, underscores the need to consider contaminant profiles in the treatment design of planned and unplanned reuse requirements to minimize potential effects to groundwater/surface-water quality or toxicological effects on plants and animals.^35−37^ There is a lack of field-scale information on the sources and fate of contaminants that occur from multiple reuse water type discharges to a surface-water system. Investigations into the mobilization and transport of WWTP-derived contaminants from rain-induced agricultural runoff from crop fields receiving effluent irrigation is important as there is mounting pressure to implement wastewater reuse to support freshwater supplies.^38^

To address these critical knowledge gaps, this study was designed within a single municipal-watershed system to (1) investigate inorganic and organic-chemical compositions, concentrations, and load contributions from three potential reuse waters (WWTP effluent, stormwater, and agricultural runoff) discharged to surface water and (2) determine if water-quality effects are observed in rain-induced runoff from a WWTP-effluent irrigated field compared to an agricultural field without such irrigation. In total, samples from five water types including surface water, effluent-irrigation for corn, urban stormwater, rain-induced runoff from an effluent-irrigated corn field (I-Ag), and rain-induced runoff from a nonirrigated corn field (NI-Ag) were analyzed. In addition, one composite at-harvest corn-plant samples were collected from each field. Water samples were analyzed for concentrations of 643 organic chemicals, 62 inorganic chemicals, and estrogenicity. Corn-plant samples were analyzed for a subset of the target organics (i.e., pharmaceuticals and PFAS). The results from this study provide the most comprehensive assessment, to date, of contaminant contributions to surface water from three reuse waters discharged from a single watershed. In addition, the results document potential downstream water-quality effects from WWTP effluent-irrigated agriculture.

## Materials and Methods

### Study Area and Sampled Sites

This study was conducted at the Oklahoma State University South-Central Research Station^28^ (SCRS) in Chickasha, OK. The SCRS is on the alluvial soils of the Washita River watershed. In 2017, the SCRS installed irrigation infrastructure to supply Category 3 reclaimed WWTP effluent^39^ from the City of Chickasha WWTP for sprinkler center-pivot irrigation on fields at rates as much as 2840 L/m (liters/minute). The WWTP was designed to receive an average daily flow of 17 million L/day for biological treatment using activated sludge and chlorination. Treated effluent irrigation was supplied (and sampled) at the downstream end of the chlorine-treatment basin prior to de-chlorination with sulfur dioxide. To characterize source-effluent irrigation, 24-h composite WWTP-effluent samples were selectively collected at times of irrigation to fields at the SCRS. The stormwater site was in conveyance infrastructure that discharged urban stormwater from 1700 hectares (19% impervious and 81% of mixed-urban area) of municipal infrastructure from the City of Chickasha.^40^

For the two agricultural field sites (I-Ag, 3.6 hectares; and NI-Ag, 4.9 hectares), farm management practices and input variables such as seed variety (Hoegemeyer-8511AML corn, 50,656 seeds/hectare), planting date (April 1, 2019), fertilizers (nitrogen, 133 kg/hectare; phosphorus, 52 kg/hectare), and herbicides (atrazine, 1.8 kg/hectare; S-metolachlor, 1.3 kg/hectare; and glyphosate, 2.4 L/hectare), and harvest date were identical during the 2019 growing season. Crop-growth stages, crop water-use requirements, and actual soil-water content were monitored by SCRS staff throughout the 2019 growing season and were used to initiate and schedule effluent irrigation at the I-Ag field. The NI-Ag field did not receive irrigation.

Composite above-ground corn-plant samples were collected at harvest from the I-Ag and NI-Ag fields as this was the portion of the corn plant that was harvested for feedstock. The surface-water site was on the Washita River 1 km upstream from the other sampled site discharge locations (Figure SI-1). To evaluate contaminant contributions to surface water from the discharge events of the three reuse waters, surface-water-grab samples were collected on the same days that rain-induced stormwater or agricultural runoff samples were collected. In total, 15 composite water samples (3 WWTP effluent, 2 stormwater, 6 agricultural [three from each of I-Ag and NI-Ag fields], and 3 surface water) and 2 composite at-harvest corn-plant samples were collected (Table SI-1). Detailed information on methods used to measure flow rates and collect composite-based samples are provided in the Supporting Information.

### Analytical Methods

Water sample analyses included quantification of 242 pesticides;^41,42^ 107 pharmaceuticals;^43^ 53 household/industrial chemicals;^44^ 58 halogenated chemicals,^13^ 48 semivolatile chemicals,^45^ 46 hormones,^14^ 34 PFAS;^46^ 33 antibiotics,^47^ 22 disinfection byproducts (DBPs),^48^ and nonvolatile dissolved organic carbon (NVDOC).^49^ Pesticide, pharmaceutical, hormone, antibiotic, DBP, and NVDOC analyses were conducted on filtered samples, whereas all other organic analyses were conducted on unfiltered samples. Water samples also were analyzed for 62 inorganic chemicals/parameters, including nutrients,^45,50,51^ alkalinity, anions, cations, trace elements (filtered), and rare-earth elements (filtered).^52^ Unfiltered methods were used, when possible, as filtered-based methods only provide dissolved concentrations and thus, likely provides an underestimation of total concentrations that were present. Filtered water-sample extracts^53^ were analyzed for total estrogenicity using the bioluminescent yeast estrogen screen.^54,55^ In addition, at-harvest corn-plant samples from the I-Ag and NI-Ag field were analyzed for a subset of organic chemicals that included 91 pharmaceuticals, 28 PFAS, and N,N-diethyl-meta-toluamide (DEET; methods described in the Supporting Information). All analytical organic and inorganic results are provided in the Supporting Information and the companion data release.^53^

### Quality Assurance

Quality assurance (QA) samples consisted of laboratory reagent-water blanks and spikes, and two field-equipment blanks (Tables SI-2–SI-4). No organic chemicals were detected in reagent-water blanks above established long-term reporting levels (RL). Organic and inorganic detections that were less than field blank sample concentrations were reported as non-detections and the RL was set at 2 times the concentration of the field blank sample. Field blank concentrations for acetophenone, DEET, and zinc had field blank concentrations that exceeded their long-term RLs (400 ng/L, 40 ng/L, and 0.2 μg/L, respectively). All acetophenone, DEET, and zinc data were retained in this paper, but the RLs were raised (1,226 ng/L, 88 ng/L, and 8.4 μg/L respectively). Overall median recoveries for laboratory reagent-spike samples ranged from 90 to 112% for target-organic methods (Table SI-5). Median recoveries for isotope-dilution standards and surrogate standards ranged 71–102% for target-organic methods.

## Results and Discussion

### Comparison of Chemistries in Three Potential Reuse Source Waters

**Figure 1:**
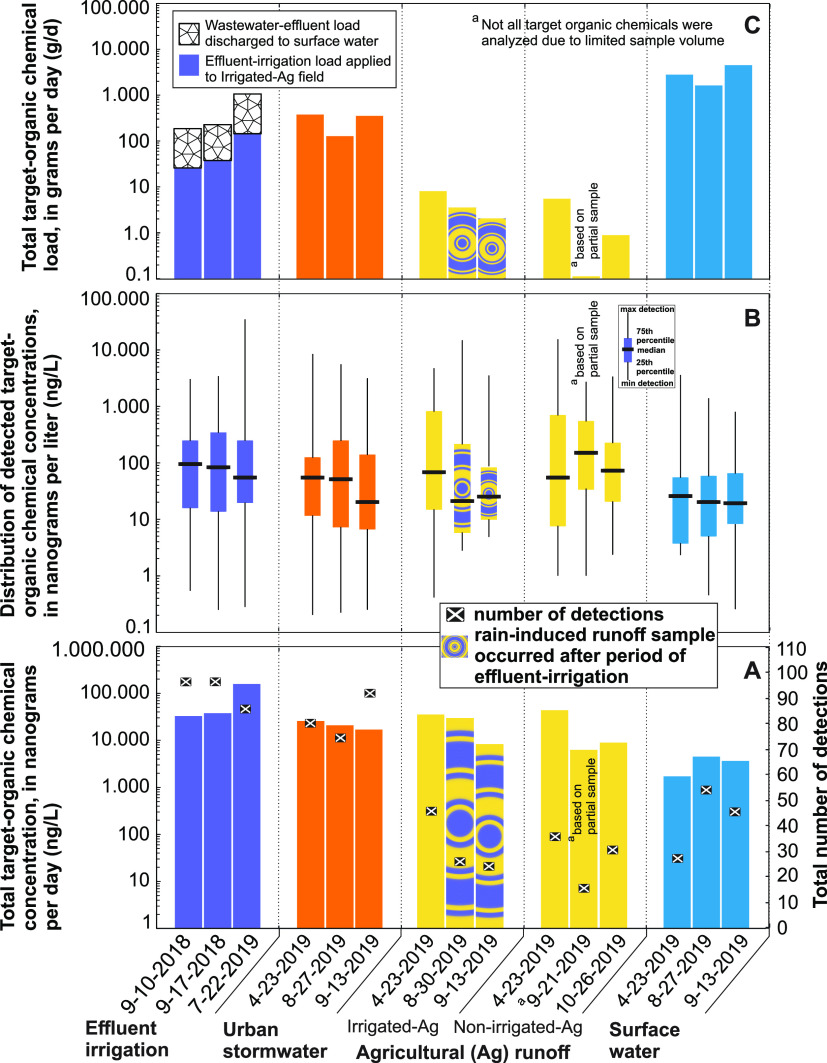
Total number of detections and concentrations (A), distribution of concentrations (B), and loads (C) for detected target-organic chemicals in samples of effluent irrigation, and rain-induced runoff of urban stormwater, agricultural fields, and surface water.

The total number of organic chemicals varied substantially among the three reuse water types (WWTP effluent, urban stormwater, and agricultural runoff) with 140 detected in WWTP effluent (hereinafter referred to as effluent), 121 in urban stormwater, and 58 in agricultural runoff samples (Table SI-6). For the 641 target-organic chemicals analyzed, 222 (34%) were detected in at least one reuse sample (Table SI-6), with 421 (66%) not detected in any sample (Table SI-7). The number of individual organic chemicals detected per sample also varied substantially between reuse types (Figure 1A and Table SI-8). Individual concentrations spanned 6 orders of magnitude across all reuse samples (from 10s of ng/L to 100s of μg/L; Figure 1B). Of the total target-organic detections (694 total) and concentration (429,000 ng/L) across all 12 reuse samples, effluent accounted for 40% of all detections and 54% of the total target-organic concentration (TCON; Figure SI-2). Urban stormwater accounted for 35% of all detections and 15% of the total target-organic concentration, whereas agricultural runoff accounted for 13% (I-Ag) and 12% (NI-Ag) of detections and 17% (I-Ag) and 14% (NI-Ag) of the total concentration. Total NVDOC concentrations were generally similar across all reuse samples and ranged in concentration from 6.5 to 23 mg/L (as carbon) in effluent, 8.2 to 12.5 mg/L in stormwater, and 8.4 to 38 mg/L in agricultural runoff (Table SI-6).

#### Pharmaceuticals

**Figure 2:**
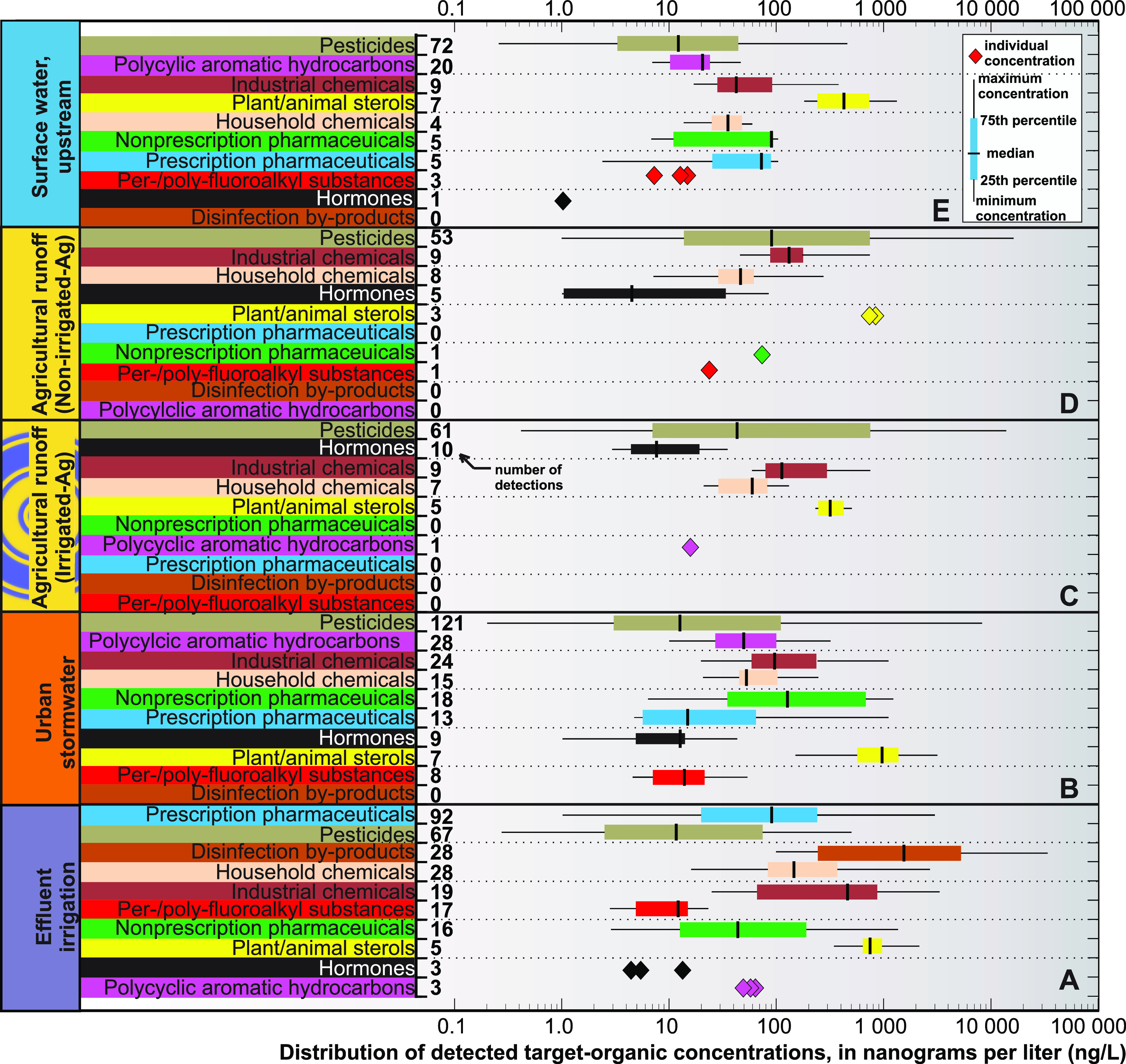
Distribution of detected target-organic chemicals in wastewater-effluent irrigation: (A) urban stormwater runoff, (B) agricultural runoff from field receiving effluent irrigation, (C) agricultural runoff from field that did not receive irrigation, and (D) surface water samples collected upstream of effluent, urban stormwater, and agricultural runoff discharge locations, sorted from top to bottom by decreasing number of total detections for a given organic-chemical class.

Prescription pharmaceuticals (P-Pharms) were detected more frequently in effluent than in urban stormwater or agricultural runoff (Figure 2). There were 42 unique P-Pharms detected in effluent that ranged in concentration from 1.1 to 3000 ng/L, accounting for 93% (23,900 ng/L) of total P-Pharms concentration across all reuse samples (Table SI-8 and Figure SI-3). Of the 42 P-Pharms detected, 36 were frequently detected (≥2 of 3 samples) in effluent. Metformin (anti-diabetic) was frequently detected in both effluent and stormwater. Metformin was detected at concentrations as large as 857 ng/L in effluent and 1020 ng/L in stormwater. Guanylurea (metformin transformation product) was frequently detected in effluent at concentrations as large as 3000 ng/L. Guanylurea is commonly detected in effluents and surface waters, with concentrations that often exceed the parent compound metformin.^56,57^ Metformin and guanylurea are prevalent environmental contaminants with documented deleterious effects to fish at low μg/L concentrations for metformin and ng/L concentrations for guanylurea.^58−64^ Maximum metformin concentrations in effluent and stormwater in our study were similar to concentrations in previous studies of municipal effluents (2580 ng/L)^65−67^ and stormwater (1260 ng/L).^13,62^

Although it was expected that P-Pharms would be more abundant in effluent, nonprescription pharmaceuticals (NP-Pharms) were detected more frequently and at greater concentrations in stormwater. There were 18 detections for 11 NP-Pharms in stormwater that accounted for 60% (6280 ng/L) of the total NP-Pharms concentration across all reuse samples, whereas there were 16 detections in effluent for 7 NP-Pharms that accounted for 39% (4140 ng/L) of the total NP-Pharms concentration (Figure SI-3). Of the 11 NP-Pharms detected in stormwater, four were frequently detected and included maximum concentrations of acetaminophen (1180 ng/L), caffeine (977 ng/L), nicotine (800 ng/L), and cotinine (60 ng/L). Concentrations of acetaminophen in our study were similar to concentrations observed in a previous study of stormwater, whereas concentrations of caffeine, nicotine, and cotinine in our study were found to be substantially less than concentrations reported in the previous study.^13^ Of the seven NP-Pharms detected in effluent, all seven were frequently detected and ranged in concentration from 3.5 ng/L (loratadine) to 1350 ng/L (fexofenadine).

#### Disinfection Byproducts

Although P-Pharms were the most frequently detected chemicals in effluent (33% of detections) they only accounted for 10% of the total target-organic concentration. In contrast, DBPs only accounted for 10% of total detections but 73% of the total concentration (Figure SI-4). DBPs are formed when chlorine and other disinfectants are used to reduce pathogen risk and waterborne disease outbreaks.^48^ The use of chlorine for disinfection is known to produce DBPs with concentrations that can have adverse effects on human health.^69^ There were 13 DBPs detected across all effluent samples with variable concentrations; total concentrations ranged from 8180 to 153,000 ng/L (Figure 2A and Table SI-8). While DBPs were ubiquitous in effluent, no DBPs were detected in stormwater and agricultural runoff. Concentration sum of chloroform, bromodichloromethane, dibromochloromethane, and bromoform (trihalomethanes, ranged from 7540 to 87,200 ng/L) in our study were similar to concentrations in a previous study of municipal effluents (2000–57,000 ng/L).^70^ Although there are regulations for some DBPs (e.g., 80,000 ng/L of trihalomethanes) for human exposure in drinking water,^71^ potential adverse effects to human and aquatic health from smaller concentrations of regulated DBP and exposures to the majority of unregulated DBP are currently unknown and constitute an important research gap.^72^

#### Household and Industrial Chemicals

Household chemicals (H-Chems) were detected more frequently and at greater concentrations in effluent than in stormwater or agricultural runoff. In effluent, there were 12 H-Chems that ranged in concentration from 16.6 ng/L (methyl-triclosan) to 2840 ng/L (acetophenone) and accounted for 84% (12,200 ng/L) of the total H-Chems concentration across all reuse samples (Figure SI-3). Of the 15 H-Chems detected across all reuse samples, nine were frequently detected in effluent, four in stormwater (benzophenone, camphor, DEET, and tri(2-chloroethyl) phosphate), and three in agricultural runoff (benzophenone, methyl salicylate, and camphor). Maximum individual concentrations were generally 1–2 orders of magnitude greater in effluent than in stormwater and agricultural runoff. Benzophenone was frequently detected across all reuse samples, ranging from a concentration of 390 ng/L in effluent to 50 ng/L in stormwater. The global usage of benzophenone in a wide array of food products, plastics, packaging materials, personal-care products, and pharmaceuticals constitutes a continuous source to the environment and a concern for negative health effects to aquatic organisms.^73−75^

Of the 14 industrial chemicals (I-Chems) detected across all reuse samples, eight I-Chems were frequently detected in stormwater, six in effluent, and four in agricultural runoff. Although I-Chems were detected more frequently in stormwater, concentrations were generally greater in effluent. I-Chems in effluent ranged in concentration from 26 ng/L (1,4-dichlorobenzene) to 3230 ng/L (phenol), accounting for 60% (12,500 ng/L) of the total I-Chems concentration across all reuse samples. In our study, no concentrations of any household or industrial chemicals exceeded a predicted toxicity-value concentration for lethal effects in aquatic organisms.^76,77^

#### Per-/Polyfluoroalkyl Substances

PFAS were detected more frequently in effluent (17 detections from nine PFAS) than in stormwater (eight detections from five PFAS) and agricultural runoff (one detection of perfluorobutanoate, 25 ng/L). The nine PFAS detected in effluent ranged in concentration from 3.0 to 23 ng/L (Figure 2A), accounting for 56% of the total PFAS concentration (371 ng/L) across all reuse samples (Figure SI-3). Although PFAS were detected more frequently in effluent with greater overall total PFAS concentration compared to the other reuse samples, the five PFAS detected in stormwater had concentrations that ranged from 4.7 to 51 ng/L, accounting for 38% (139 ng/L) of the total PFAS concentration. PFAS have been shown to cause disruption to key cellular functions and can cause negative biological effects when animals and humans are exposed to PFAS.^78−81^ Exposure to PFAS at low concentrations is of environmental concern because they are largely resistant to biotic transformations and exhibit bioaccumulation potential.^13^ Perfluorooctanesulfonate (PFOS) linear^82^ was frequently detected in effluent and stormwater at concentrations that ranged from 12 to 13 ng/L in effluent and 15 to 51 ng/L in stormwater. There are few data on the occurrence of PFAS in stormwater, although one recent study documented low concentrations (<2.0 ng/L) of PFOS in stormwater.^83^ Four additional PFAS were frequently detected in effluent with maximum concentrations of perfluoropentanoate (PFPeA, 23 ng/L), perfluorobutanesulfonate (PFBS, 15 ng/L), perfluorohexanoate (PFHxA, 18 ng/L), and perfluorononanoate (PFNA, 4.2 ng/L). Concentrations of PFBS and PFHxA in effluent for our study were similar to concentrations reported in a previous study of effluents.^67^ Perfluorooctanoate (PFOA) was detected only once (7.3 ng/L) in our study, but a previous study documented frequent PFOA concentrations in effluents with concentrations as large as 1400 ng/L.^67^ In our study, branched PFOS was only detected in stormwater at concentrations that ranged from 7.5 to 13 ng/L. No concentrations of any PFAS detected in effluent, stormwater, and agricultural runoff samples in our study exceeded published lowest-observed effect levels (NOEL) for aquatic organisms.^76^

#### Polycyclic Aromatic Hydrocarbons (PAHs)

No PAHs were frequently detected in agricultural runoff and were minimally detected in effluent, with only anthraquinone, an additive in paper, detected in all three effluent samples (concentrations ranged from 52 to 64 ng/L). There were 10 PAHs frequently detected in urban stormwater, with concentrations ranging from 10 to 310 ng/L (Figure 2A). There was a total of 28 PAH detections in stormwater samples that accounted for 93% of the total PAH concentration (2350 ng/L) across all reuse samples. Of the 10 PAHs frequently detected in stormwater, benzo[*a*]anthracene, benzo[*a*]pyrene, benzo[*b*]fluoranthene, benzo[*k*]fluoranthene, chrysene, and indeno[1,2,3-*cd*]pyrene are designated as probable human carcinogens.^84^ Of the total 28 PAH detections in stormwater, 12 exceeded human health ambient water-quality criteria concentrations.^85^ In a previous national study of stormwater, PAHs also were frequently detected but at greater individual concentrations (10 s to 10,000 ng/L).^13^ This previous national study documented increasing trace-organic contributions (including PAHs) with increasing drainage area, impervious surfaces, and developed high-intensity land-use/land-cover (LULC).

#### Pesticides

Pesticides were detected more frequently in urban stormwater compared to effluent and agricultural runoff. In total, there were 56 pesticides (121 detections) in stormwater, 41 pesticides (67 detections) in effluent, followed by agricultural runoff from the I-Ag field with 37 pesticides (61 detections) and from the NI-Ag field with 34 pesticides (53 detections). Although pesticides were detected less frequently in agricultural runoff than in stormwater and effluent, concentrations were substantially greater in agricultural runoff (Figure 2 and Table SI-8). In agricultural runoff, maximum pesticide concentrations ranged from 14,000 ng/L in I-Ag samples to 17,800 ng/L in NI-Ag samples. Total pesticide concentration in I-Ag (68,500 ng/L) and NI-Ag (51,100 ng/L) accounted for 74% of the combined total pesticide concentration across all reuse samples.

Extensively used agricultural herbicides glyphosate, 2,4-Dichlorophenoxyacetic acid (2,4-D), metolachlor, and atrazine were frequently detected in agricultural runoff and stormwater, but not in effluent. Maximum concentrations of glyphosate (14,000 ng/L), 2,4-D (12,900 ng/L), and metolachlor (2990 ng/L) in agricultural runoff were substantially greater than concentrations in stormwater (8200, 3680, and 87 ng/L, respectively). Three transformation products (TPs) of atrazine (didealkylatrazine, deethylatrazine, and 2-hydroxy-4-isopropylamino-6-amino-s-triazine) and four TPs of metolachlor (dechlorometolachlor, hydroxymetolachlor, metolachlor SA, and metolachlor OA) also were frequently detected in agricultural runoff, but not in effluent or stormwater. Previous research indicated that TPs may be contributing substantially more instream toxicity than previously understood.^86^ Although concentrations of glyphosate, 2,4-D, and atrazine in our study did not exceed chronic aquatic life benchmark (ALB) concentrations, 50% (three detections) of the metolachlor concentrations in agricultural runoff exceeded the 100 ng/L ALB concentration for invertebrates.^87^ In addition, 67% (four detections) of metolachlor OA concentrations in agricultural runoff samples in our study exceeded the 4200 ng/L concentration shown to have chronic health effects to aquatic organisms.^88^

There were seven pesticides (six insecticides and one herbicide) that were frequently detected in effluent and stormwater, but not in agricultural runoff. The frequently detected banned insecticides, technical chlordane (*cis*-chlordane, trans-chlordane, trans-nonachlor) and dieldrin were detected at greater concentrations (0.6–5.5 ng/L) in effluent than in stormwater (0.5–0.9 ng/L). Although not detected in stormwater and agricultural runoff, the restricted-use insecticide 2,4,6-trichlorophenol was detected in every effluent sample with concentrations as large as 120 ng/L. The frequent detection of such legacy and restricted-use insecticides illustrates the importance of continued monitoring and management of persistent compounds that are no longer used but may still pose aquatic or human health risks.^11,89^ Fipronil (insecticide), fipronil sulfide (fipronil TP), and bromacil (herbicide) also were frequently detected in both stormwater and effluent, but not in agricultural runoff. Both fipronil and fipronil sulfide were detected at a greater maximum concentration (60 and 6.1 ng/L, respectively) in effluent than in stormwater (3.5 and 0.5 ng/L, respectively). All fipronil concentrations in effluent exceeded the 11 ng/L ALB concentration.^87^ Bromacil was detected at a substantially greater maximum concentration (2090 ng/L) in stormwater than in effluent (313 ng/L).

Previous studies have documented increasing insecticide detection in effluent and stormwater, attributed to increased home and garden use, at concentrations that can exceed those in agriculture.^13,68,90−93^ Herbicide transformation products aminomethylphosphonic acid (AMPA) and 2-Hydroxyatrazine (OIET) were detected at greater concentrations in agricultural runoff, whereas imidacloprid was detected at a greater concentration in effluent. Imidacloprid concentrations were as large as 218 ng/L in effluent, 28 ng/L in agricultural runoff, and 7 ng/L in stormwater. The neonicotinoid insecticide clothianidin was detected at concentrations as large as 1050 ng/L in agricultural runoff and 235 ng/L in effluent. All detected imidacloprid and clothianidin concentrations in effluent exceeded the chronic ALB concentrations for imidacloprid (10 ng/L) and clothianidin (50 ng/L) exposure to aquatic organisms.^87^ Even though imidacloprid and clothianidin are widely used in agriculture, previous research has implicated WWTPs as a point source for imidacloprid and clothianidin.^94^ In addition, a previous study documented comparable chronic toxicity to aquatic organisms from individual exposure concentration ranges from 17 to 290 ng/L for imidacloprid and 10–380 ng/L for clothianidin.^95,96^ An additional 25 pesticides were frequently detected in stormwater, but not in effluent or agricultural runoff. Concentrations of these 25 frequently detected pesticides in stormwater had concentrations that generally ranged from ∼10 to 100 ng/L (Table SI-6).

#### Estrogenicity and Hormonally Active Chemicals

Effluent, stormwater, and agricultural runoff are considered major sources of EDCs to aquatic environments.^8,19,97−99^ In the current study, the total concentration of estrogenic compounds in reuse samples was estimated as 17β-estradiol equivalents (E2Eq) using a bioluminescent yeast estrogen screen.^54^ Current effects-based trigger (EBT) values for estrogens are defined by a range of 0.1–0.5 ng/L E2Eq.^100^ Estrogenic activity was measurable in the majority (73%) of all reuse samples (Table SI-6), with the largest E2Eq concentrations measured in stormwater (0.756–1.01 ng/L), followed by agricultural runoff (0.22–0.46 ng/L), and effluent (<0.13–0.15 ng/L). Previous studies on endocrine disruption in fish indicated 1 ng/L E2Eq as the predicted no-effect concentration of total estrogens on fish reproduction.^101^ In our study, only 1 stormwater runoff event on 9/13/2019 exceeded the risk level for endocrine disruption (>1 ng/L E2Eq); however, 79% of samples were within or above the EBT.

Previous studies on streams have documented estrone to be detected more frequently and measured at greater concentrations than other natural estrogens.^102^ In our study, estrone was frequently detected across all reuse samples at a similar low ng/L range in effluent (3.4–5.4 ng/L), agricultural runoff (1.0–6.3 ng/L), and stormwater runoff (1.6–3.1 ng/L). Effects from exposure to estrone in streams at low ng/L (1.0–15 ng/L) concentrations have been linked to reproductive effects in aquatic organisms^102,103^ In our study, two phytoestrogens, daidzein and formononetin, were frequently detected in stormwater at concentrations as large as 14 and 42 ng/L, respectively, but not in effluent or agricultural runoff. A previous study documented an order of magnitude lower concentrations of daidzein and formononetin in urban-impacted streams and effluents.^104^

#### Inorganic Chemicals

The inorganic-chemical concentrations were generally dilute in all reuse waters. Data for these constituents, including maximum specific conductance values in effluent (1015 μS/cm), stormwater (425 μS/cm), and agricultural runoff (135 μS/cm) are shown in Table SI-9. Chloride and bicarbonate were the most abundant anions with maximum concentrations found in effluent (74.0 and 254 mg/L, respectively). The major cation composition of effluent and stormwater was dominated by sodium and calcium, whereas agricultural runoff was dominated by calcium and magnesium. Concentrations of total nitrogen were greatest in effluent (26.1 mg/L) and least in stormwater (2.5 mg/L). Total phosphorus concentrations were greatest in effluent (4.4 mg/L) and least in stormwater (0.66 mg/L). Additional results and discussion of these inorganic chemicals, including trace metals, are provided in the Supporting Information.

### Water-Quality Effects to Rain-Induced Agricultural Runoff from Wastewater-Effluent Irrigation

Source effluent-irrigation samples collected during irrigation were used to quantify the target organic-chemicals contribution to the I-Ag field that could potentially be mobilized and transported to receiving surface waters during subsequent rain-induced runoff events. For the three sampled runoff events from the I-Ag field, one runoff event was sampled prior to effluent irrigation and two runoff events were sampled after effluent irrigation. Coincident with onset of a dry period, a total of 6.3 million L of effluent irrigation was applied to the I-Ag over a 24-day period. Based on the total irrigation volume and mean organic-chemical concentrations from the source-effluent irrigation samples, an estimated target-chemicals total organic load of 491 g was applied to the I-Ag field during the 24-day effluent-irrigation period. For source-effluent irrigation, DBPs accounted for 73% (356 g) of the total target-organic-chemical load, followed by 10.3% (51 g) of prescription pharmaceuticals, industrial chemicals (31 g), household chemicals (26 g), plant/animal sterols (9.7 g), nonprescription pharmaceuticals (8.7 g), pesticides (8.3 g), PFAS (0.44 g), PAHs (0.36 g), and biogenic hormones (<0.05 g, Figure SI-5).

The total target-organic-chemical load in runoff from the I-Ag field before irrigation was 7.9 g/day, whereas the load in the two subsequent runoff events after irrigation were 3.3 and 2.0 g/day, respectively (Figure 1C and Table SI-8). The total pesticide load in I-Ag runoff before irrigation was 7.5 g/day, followed by the load from the first subsequent runoff event after irrigation (3.1 g/day), and the second runoff event after irrigation (1.8 g/day), which accounted for 95, 93, and 93%, respectively, of the total target-organic-chemical load in I-Ag samples. The decreasing load amounts, but at similar load proportions, is an indication that the pesticides (2,4-D, atrazine, S-metolachlor, and glyphosate) applied to the I-Ag field at planting, contributed substantial organic loads via runoff that were “flushed” from the I-Ag field during subsequent rain-induced runoff events. Across all I-Ag runoff samples, 82% of the total pesticide load was composed of the parent compounds and TPs of 2,4-D, atrazine, glyphosate, and metolachlor, which were either not detected or detected generally at low concentrations (<50 ng/L) in source-effluent irrigation. Overall, the 491 g total target-organic-chemical load contribution from effluent irrigation to the I-Ag field did not produce substantial dissolved organic-contaminant contributions in subsequent rain-induced runoff events and is an indication of effective natural attenuation processes that reduced or transformed organics, partitioning into soils, or uptake by plants. A limitation of this study is that analysis of target-organic contaminants in soils from our fields was not included.

Comparative analysis of mean concentrations of individual organic chemicals in runoff from I-Ag and NI-Ag fields and detections unique to effluent irrigation (not detected in NI-Ag runoff), revealed eight organic chemicals that were frequently detected in source-effluent irrigation and I-Ag runoff. Of the eight suspect contaminants, two organic chemicals (estrone and imidacloprid) had differences in individual concentrations that were apparent between agricultural runoff from the I-Ag and NI-Ag fields. In the sample collected prior to effluent irrigation, estrone was not detected in runoff from the I-Ag field but was detected at concentrations of 3.9 and 6.3 ng/L in samples from two subsequent runoff events collected after effluent irrigation. Estrone was frequently detected in source-effluent irrigation at concentrations that ranged from 3.4 to 5.4 ng/L, similar to the concentrations in I-Ag runoff. Estrone is an endocrine-disrupting chemical that is widely recognized as a concern to aquatic organisms at low ng/L concentrations and has been shown to have negative reproductive effects.^102,103^ Evidence indicates that the role of estrone as an EDC has been greatly underestimated.^108^ Owing to incomplete hormone removal (range 65–95%), WWTPs are a substantial pathway of estrone into aquatic ecosystems.^10^ Previous studies have documented substantially greater estrone concentrations in rain-induced agricultural runoff from bovine-waste amended fields^109^ and similar concentrations in poultry-waste^109^ and municipal biosolids^26^ amended fields. A previous study also documented estrone at concentrations as large as 50 ng/L in excess runoff from WWTP-effluent irrigation but not in rain-induced runoff.^15^ Imidacloprid was ubiquitous in source-effluent irrigation and I-Ag runoff samples but was not detected in NI-Ag runoff samples. Imidacloprid concentrations were an order of magnitude greater in effluent irrigation (124–218 ng/L) than in I-Ag runoff (7.0–28 ng/L). The imidacloprid concentrations in effluent irrigation in our study were similar to those reported in a study of effluent from 13 WWTPs in the US.^110^ Imidacloprid is a neonicotinoid insecticide formulated to disrupt neural transmission in the central nervous system of insects.^111,112^ Effects of imidacloprid and other neonicotinoids can substantially alter ecosystem structure and function because of their effects on non-target insects and aquatic organisms.^113^ For this study, the estrone and imidacloprid concentrations in effluent irrigation and resulting concentrations in rain-induced runoff from the I-Ag field exceeded ALB or no-health-effect concentrations, raising concerns for aquatic species in receiving surface waters.

There is evidence for potential human and animal exposures from food consumption of plants that have taken up WWTP-derived contaminants, underscoring the need to evaluate other contaminant exposure pathways and antibiotic resistance.^21−24^ To help address this important topic, we assessed if effluent irrigation to corn influenced contaminant uptake through the collection and analyses of above-ground corn plants (e.g., stem, leaf, and kernel) collected at harvest from the I-Ag and NI-Ag fields. Such above-ground corn-plant samples were collected because that was the portion of the plant being fed to cattle following harvest. The corn-plant samples were analyzed for 91 pharmaceuticals, 28 PFAS, and DEET. Many of our target organics (e.g., pharmaceuticals) were measured in filtered water samples and we were not able to measure for our complete suite of 641 organics in plant tissue, which limited our interpretations. Nevertheless, of the 91 pharmaceuticals analyzed in the two at-harvest corn-plant samples, two prescription antibiotics (norfloxacin and ciprofloxacin) were detected at concentrations of 36 and 70 ng/g, respectively, in corn-plant samples from the I-Ag field but not in corn-plant samples from the NI-Ag field (Table SI-10). Previous research has documented maximum antibiotic residue concentrations of norfloxacin and ciprofloxacin in vegetables and cereals that ranged from 0.27 to 658 and 2.5 to 39.0 ng/g, respectively.^29,114^ In a comprehensive study of human dietary intakes of antibiotic residuals in water and food products, consumption of plant-derived food resulted in the greatest potential health risk from daily intake rates of ciprofloxacin and norfloxacin.^29^ DEET also was detected in corn-plant samples from the I-Ag field (3.3 ng/g) but not in corn-plant samples from the NI-Ag field. Ciprofloxacin concentrations in source effluent-irrigation samples ranged from 12 to 15 ng/L and DEET concentrations ranged from 100 to 212 ng/L. Norfloxacin was not detected in source effluent-irrigation samples. Ciprofloxacin and norfloxacin are known to remain active and select for microbial resistance in soils,^115^ for sorption to soils and organic matter,^116,117^ and for uptake in plants.^118,119^ Therefore, low detection concentrations or lack of detection (e.g., norfloxacin) in filtered source effluent-irrigation and agricultural runoff samples in our study was not surprising and documents a limiting factor of our study; that two different methods (a total-based method for plant tissue and dissolved-based method for water) were used to measure ciprofloxacin and norfloxacin. PFAS were detected at low concentrations in source-effluent irrigation, but not in corn-plant samples from the I-Ag and NI-Ag fields.

Measurements of rare-earth elements (REEs), boron (B), and chloride (Cl^–^) in agricultural runoff from fields that have received effluent irrigation can be useful tracers of municipal waste and can aid in the identification of natural versus municipal contaminant sources.^13^ Anthropogenic gadolinium (Gd_anthro_) is a synthetic organic Gd complex used in medical diagnostics since 1988,^105^ and a Gd_anthro_/Gd_background_ ratio >1.5 is commonly observed in municipal wastewater effluents^106,107^ Not surprisingly, effluent used as irrigation in this study exhibited a substantial Gd ratio anomaly (6.9). No substantial Gd ratio anomalies (>1.5), however, were present in agricultural runoff samples from I-Ag field before effluent irrigation (Gd, 1.1) and after effluent irrigation (Gd, 1.2 and 1.2, Table SI-9). Although B and Cl^–^ concentrations were substantially greater in effluent (maximum; 0.39 and 74 mg/L, respectively) than in agricultural runoff (maximum; 0.04 and 2.1 mg/L, respectively), no apparent differences were present in runoff from the I-Ag field before and after effluent irrigation or between runoff samples from the I-Ag and NI-Ag fields. Prior to our study, effluent irrigation had not routinely been applied to the I-Ag field. Comparative analysis of the other inorganic chemicals did not reveal any observable differences in concentrations in runoff samples pre- and post-effluent irrigation of the I-Ag field or in runoff samples from I-Ag and NI-Ag fields.

### Organic Chemistry of Receiving Surface Water and Loadings from Three Discharged Reuse Waters

The three surface-water samples collected upstream from all reuse water discharge locations (Figure SI-1) were characterized by fewer overall organic detections (28–53) than those in effluent and stormwater, with substantially lower concentrations than those in effluent, stormwater, and agricultural runoff (Figure 2 and Table SI-8). Pesticides and PAHs were the most frequently detected organic chemicals in surface water, accounting for 57 and 16%, respectively, of the total organic detections in surface water, followed by household/industrial chemicals (10%), pharmaceuticals (8%), plant/animal sterols (∼5.6%), PFAS (∼2.4%), and hormones (∼<0.1%). In total, there were 23 pesticides and 10 PAHs frequently detected (≥2 of 3 samples) in surface water at substantially lower concentrations than in stormwater. Maximum total target-organic concentration in surface-water samples (4417 ng/L) was 1–2 orders of magnitude lower than in effluent (165,000 ng/L), stormwater (25,800 ng/L), and agricultural runoff (44,200 ng/L). Metformin was detected in every surface-water sample at concentrations that ranged from 74 to 105 ng/L and PFPeA was frequently detected at concentrations that ranged from 7.2 to 16 ng/L. Previous studies have documented that metformin is a prevalent contaminant in surface waters at concentrations ranging from 1 to 3000 ng/L.^63,120^ PFPeA, one of several PFAS that are widely used in consumer products for their nonstick and stain resistance properties, has been documented in streams at concentrations ranging from 1.2 to 84 ng/L.^121^ Overall, organic concentrations in samples of surface water collected upstream from reuse discharge locations in our study were similar to concentrations reported in a previous study of US streams.^122^

The total target-organic-chemical load from surface-water samples collected upstream from effluent, stormwater, and agricultural runoff discharges was calculated to assess load contributions from daily effluent and total storm-event loadings from stormwater and agricultural runoff. Because of the substantially greater surface-water flow volumes, the total surface-water organic load was greater than loads from effluent, stormwater, and agricultural runoff (Figure 1C). For all sampled events, effluent contributed ∼0.6% (19.2 million L) of additional discharge to surface water. Discharge contribution to surface water from stormwater (∼1.2%, 41.1 million L) was more than 2 times that of effluent. Agricultural runoff contributed substantially less discharge volume (∼0.02%, 0.81 million L) to surface water for the sampled events. Although the total discharge volume (61.1 million L) from all effluent, stormwater, and agricultural runoff events only accounted for 1.8% of the total surface-water discharge volume (3.33 billion L), the total target-organic-chemical load to surface water from effluent accounted for 13% (1500 g) of the organic load across all sites (11,800 g), followed by stormwater (7.6%, 891 g) and agricultural runoff (<0.2%, 19.6 g).

Combined total loads to surface water from DBPs (1110 g), prescription pharmaceuticals (151 g), industrial chemicals (80 g), and household chemicals (78 g) were largest from effluent, whereas total loads to surface water from pesticides (514 g), plant/animal sterols (145 g), nonprescription pharmaceuticals (91 g), PAHs (26 g), and biogenic hormones (1.8 g) were largest from stormwater (Figure SI-6 and Table SI-8). A previous national study of urban stormwater documented a significant positive correlation between trace-organic contributions (including PAHs and pesticides) and drainage area, impervious surfaces, and developed high-intensity LULC. Since our sampled stormwater site received urban runoff from a large (17 km) drainage area that consisted of 19% impervious and 81% mixed-urban LULC,^40^ it was not surprising that in our study we also would have large PAH and pesticide contributions. In addition, temporally increasing pesticide detection has been reported in urban streams and attributed to increased home and garden use in the urban landscape.^91^ Previous studies of effluent and stormwater have documented overall smaller prescription pharmaceutical loadings from stormwater.^9,13,110^ Although the overall prescription pharmaceutical load from effluent was greater than from stormwater (34 g) in our study, the individual total metformin load to surface water from stormwater (22 g) was more than 2 times greater than the metformin load contribution from effluent (9.8 g).

Loadings of PAHs and pesticides in our study were similar to previous studies that reported substantially greater loadings in stormwater than those from effluent.^9,13,110^ Although previous studies have shown loadings of NP-Pharms (e.g., acetaminophen, caffeine, lidocaine, and nicotine) to be similar from effluent and stormwater,^13^ in our study, loadings from nonprescription pharmaceuticals (91 g) were more than 3 times greater in stormwater than from effluent (26 g). In addition, the frequently detected nonprescription pharmaceuticals (acetaminophen, caffeine, cotinine, and nicotine) in stormwater contributed substantially greater cumulative contaminant loads (77 g) to surface water than effluent (0.2 g). Compared to effluent and stormwater, agricultural runoff exhibited small organic-load contributions, owing to smaller discharge volumes from the smaller drainage areas and greater soil water-infiltration capacity. Although the total organic load in agricultural runoff was substantially smaller than effluent or stormwater, total pesticide load contributions from agricultural runoff (18 g) were substantial, being generally similar to pesticide loads from effluent (25 g). Results from our study document substantial organic-chemical contributions to surface water from effluent (DBPs, prescription pharmaceuticals, industrial chemicals, and household chemicals), stormwater (pesticides, nonprescription pharmaceuticals, PAHs, and biogenic hormones), and agricultural runoff (pesticides). Excluding DBPs, episodic storm-event organic concentrations and loads from stormwater were comparable to and often exceeded those of daily effluent discharges (Figure SI-6).

### Implications for Environmental Receptors and Reuse

Results from our study were consistent with previous findings that potential reuse waters (WWTP effluent, stormwater, and agricultural runoff) contain extensive and unique mixtures of organic chemicals that are transported to receiving surface waters through continuous discharge or episodic storm-event discharges. Many of the detected chemicals are known to persist in the environment and, therefore, are priority considerations for the development of reuse best practices and of planned or unplanned reuse requirements.^123^ The required filtering of water samples for some analytical methods (e.g., pesticides and pharmaceuticals) likely provides an underestimation of total concentrations being transported during rain-induced runoff. Total organic-chemical concentrations and loads from stormwater runoff events were comparable to and often exceeded those of daily WWTP discharges. The chemicals detected in sampled reuse waters are of concern in terms of potential biological exposures to terrestrial and aquatic organisms, water-quality effects to receiving surface and groundwaters, and overall ecosystem health because many of the chemicals are known carcinogens (e.g., PAHs), designed bioactives (e.g., pesticides and pharmaceuticals), or hormonally active (e.g., PFAS and hormones).^122^ Environmental health effects of complex organic mixtures at low concentrations, as seen in our study, are poorly understood, but a range of potential effects are possible even when chemicals determined not to have individual effects are present in mixtures at low ng/L concentrations.^58,124−126^ With a few exceptions, the contaminants found in effluent irrigation in our study were not observed in subsequent rain-induced runoff. This contrasts with research showing elevated contaminant concentrations in runoff when municipal biosolids are applied instead of effluent irrigation.^27^ However, there are some notable exceptions that pose potential environmental implications and health concerns for the reuse of effluent irrigation on agricultural fields, such as water-quality effects of rain-induced runoff (i.e., imidacloprid, estrone) and plant uptake (ciprofloxacin and norfloxacin). The concentration levels of ciprofloxacin and norfloxacin in at-harvest corn-plant samples in our study were similar to ciprofloxacin and norfloxacin concentrations detected in food from a human dietary intake study that indicated human consumption of plant-derived food posed substantial human health risk.^29^ Our study underscores the need for additional research to further evaluate the pathways and mechanisms of antibiotic resistance as well as the sources, transport, and fate of contaminants from other land-applied reuse materials (e.g., municipal biosolids and livestock waste) used for growing crops across all relevant environmental media (runoff, soil, plant).

## Supporting Information Available

Additional method details and sample results information (PDF)
Additional site information, quality assurance results, and all sample results (XLSX)
