# Supplementary material for: Contaminant Exposure and Transport from Three Potential Reuse Waters within a Single Watershed
Source: Environ Sci Technol. 2023 Jan 10;57(3):1353–65. doi: 10.1021/acs.est.2c07372 (PMC9878729; doi:10.1021/acs.est.2c07372)
Supplement: Supplementary file 1 — es2c07372_si_001.pdf [file es2c07372_si_001.pdf]

Supporting Information for:

Contaminant Exposure and Transport from Three Potential Re-use Waters Within a Single Watershed

Jason R. Masoner,*[A] Dana W. Kolpin,[B] Isabelle M. Cozzarelli,[C] Paul M. Bradley,[D] Brian B. Arnall,[E] Kenneth J. Forshay,[F] James L. Gray,[G] Justin F. Groves,[F] Michelle L. Hladik,[H] Laura E. Hubbard,[I] Luke R. Iwanowicz,[J] Jeanne B. Jaeschke,[C] Rachael F. Lane,[K] Richard Blaine McCleskey,[L] Bridgette F. Polite,[C] David A. Roth,[L] Michael B. Pettijohn,[E] and Michaelah C. Wilson[K]

*Corresponding Author, E-mail: jmasoner@usgs.gov
[A]U.S. Geological Survey, Oklahoma City, Oklahoma 73116, United States
[B]U.S. Geological Survey, Iowa City, Iowa 52240, United States
[C]U.S. Geological Survey, Reston, Virginia 20192, United States
[D]U.S. Geological Survey, Columbia, South Carolina 29210, United States
[E]Oklahoma State University, Stillwater, Oklahoma 74078, United States
[F]U.S. Environmental Protection Agency, Ada, Oklahoma 74820, United States
[G]U.S. Geological Survey, Lakewood, Colorado 80225, United States
[H]U.S. Geological Survey, Sacramento, California 95819, United States
[I]U.S. Geological Survey, Madison, Wisconsin 53711, United States
[J]U.S. Geological Survey, Kearneysville, West Virginia, 25430, United States
[K]U.S. Geological Survey, Lawrence, Kansas 66049, United States
[L]U.S. Geological Survey, Boulder, Colorado 80303, United States

Supporting information (SI) for this manuscript is made available in two files that contain additional site information, method details, and analyses of results. There are 14 pages of SI information included in this file (SI-Info.docx). In addition, this file includes six SI Figures (with captions) that start on page 8. There are 10 SI Tables in the corresponding SI spreadsheet (SI-Tables.xlsx). The individual SI table titles are listed on page 13 and 14 of this file and in each of the 10 table worksheets in the spreadsheet (SI-Tables.xlsx).

MATERIALS AND METHODS

Study Area

The Oklahoma State University (OSU) South-Central Research Station (SCRS) is in the northeast part of Chickasha, OK, on alluvial soils of the Washita River that consist primarily of the Dale and McClain soil series. These soils are commonly found on flood plains and terraces of Oklahoma's east-west through-flowing rivers and are among the best agricultural soils in the state because of their favorable texture and structure for plant growth (OSU, 2022).[1] Crop-growth stages, crop water-use requirements, and actual soil-water content were monitored by SCRS staff throughout the 2019 growing season and were used to initiate and schedule effluent-irrigation at the Irrigated-Ag (I-Ag) corn field.

Sampling methods

Sampling equipment, tubing, and composite-sampling containers used to collect samples were (1) cleaned in a commercial glassware-style dishwasher using tap water and anionic detergent and rinsed with tap water followed by deionized water, (2) rinsed with a 3% acetic acid rinse, and (3) air-dried and sealed in clean plastic bags. Flow-weighted composite samples were collected from each of the Irrigated-Ag, Non-Irrigated-Ag, and Urban Stormwater sites. Time-weighted (24-hour) composite samples of effluent irrigation were collected at the wastewater treatment plant from the chlorine-treatment basin prior to de-chlorination with sulfur dioxide gas. The chlorine-treatment basin provides disinfection of wastewater by providing sufficient mixing and detention time of chlorine and wastewater. All flow-weighted and time-weighted samples were collected using refrigerated-automated samplers (Teledyne, Thousand Oaks, CA). Both flow-weighted and time-weighted automated sample approaches targeted a 16-L composite sample (that consisted of 14 to 24 subsamples of 1000 mL each) collected across the entire rain-induced runoff event or over a 24-hour period (wastewater-effluent irrigation).

Automated samplers were equipped with 30-cm long silicone peristaltic-pump tubing attached to high-density polyethylene tubing, stainless steel intake screen (14 mm diameter openings, no mesh), and 17-L high-density polyethylene container. Automated samplers were programmed to perform a field rinse through the sample tubing from the intake screen to the peristaltic pump head upon initiation and prior to collection of each subsample. Composite samples were retrieved within 24 h of a sampling event and were processed and split in laboratory at the SCRS. A 14-L high-density polyethylene churn-splitter was used to split the composite sample into subsamples for specific chemical analyses following published methods.[2] For the two at-harvest plant samples, five above-ground-corn plants were collected from each of the Irrigated-Ag and Non-Irrigated-Ag fields and 1 kg each for stem, leaf, and kernel parts were sub-sampled (3 kg total), composited, and sent to SGS AXYS Analytical Services laboratory for grinding and chemical analyses for pharmaceuticals and per-/poly-fluoroalkyl substances (PFAS). All water and plant tissue samples were shipped on ice with next-day delivery. For at-harvest corn plant sampling, each corn field was split into five quadrants, and one above-ground-corn plant was collected from each of the five quadrants to provide plants for composite sampling. Details concerning unpublished methods of pharmaceutical and PFAS analyses for plant samples are provided below in “Analytical Methods” section.

Urban stormwater discharge was calculated based on pipe/channel geometry and measurements of velocity and water level recorded in 2-minute increments (750 flow module, Teledyne, Thousand Oaks, CA). Agricultural runoff discharge was calculated based on established discharge ratings for 20-degree v-notched concrete weirs and measurement of water level recorded in 2-minute increments (750 flow module, Teledyne, Thousand Oaks, CA). Estimates of discharge were downloaded from the U.S. Geological Survey National Water Information System database for nearby U.S. Geological Survey streamgage 07328100 Washita River at Alex, OK[3] and were used to estimate flow at the sampled Surface Water site. Single storm-event runoff (urban, agricultural, and surface water), daily WWTP effluent discharge volumes, and cumulative organic-chemical concentrations at sampled sites were used to calculate load estimates for target-organic chemicals.

Analytical Methods

Per-/polyfluoroalkyl substances (PFAS) in plant samples (unpublished method):

PFAS analyses for plant samples were analyzed by SGS AXYS Analytical Services under a contract with RTI Laboratories, Inc. (Livonia, MI) by using liquid chromatography tandem mass spectrometry (LC-MS/MS PFAS). Up to 5 g (dry weight) or 10 g (wet weight) was extracted and analyzed for each sample (0.5 g dry weight and 5 g wet weight for biosolids). Isotopically labeled surrogate standards were added prior to adding a methanolic ammonium hydroxide solution. The supernatants were combined, treated with ultrapure carbon powder and methanol, which was then removed by evaporation. The resulting extracts were diluted in water and cleaned up using solid-phase extraction (SPE) with a weak anion-exchange sorbent. The eluate was spiked with recovery standards and analyzed by LC-MS/MS with negative electrospray ionization. The instrument was calibrated within a linear range determined by a minimum of five calibration levels using the constant relative response factor (RRF) technique (Chakravarthy et al., 2011).[4] Reporting levels were adjusted to account for the lowest calibration level. Isotope-dilution quantification techniques were used for the calculation of results.

Pharmaceuticals in plant samples (unpublished method performed by SGS AXYS Analytical Services):

Pharmaceuticals (LC-MS/MS pharmaceuticals) analyses for plant samples were analyzed by SGS AXYS Analytical Services and divided into three groups set by laboratory-defined lists (Lists 1,4,5), processed, and reported under slightly different protocols. Solid samples were extracted by sonication in buffered or pure acetonitrile concentrated by rotary evaporation and diluted with ultra-pure water to a final volume of 200 mL; the buffered acetonitrile was acidic (pH2, Lists 1 and 5). The acidic extracts were treated with ethylenediaminetetraacetic acid (EDTA), a synthetic chelation agent. All extracts underwent sample cleanup with SPE, were spiked with recovery standards, and analyzed by LC-MS/MS with positive electrospray ionization. The instrument was calibrated within a linear range determined by a minimum of five calibration levels using linear regression (List 1) or constant RRF (Lists 4 and 5) technique. Reporting levels were adjusted to account for the lowest calibration level used to determine the calibration curve. Internal standard (List 1) or isotope-dilution (Lists 4 and 5) quantification techniques were used to calculate results.

Total estrogenicity, bioluminescent yeast estrogen screen (BLYES):

The bioassay solid-phase extraction (SPE) was developed at the U.S. Geological Survey Kansas Water Science Center, Organic Geochemistry Research Laboratory (OGRL) to provide extracts for analysis at other laboratories. Water samples were collected in 1 L amber glass bottles with Teflon lined caps, packed with ice, and shipped overnight to OGRL. After arrival at OGRL, samples were stored at 2-4 °C, filtered with a 0.7-micron glass-fiber filter within 24 hours of arrival, and extracted within 36 hours of arrival. The general concentration scheme is 1L of sample to 100 µL of extract. For the 2019 Chickasha (CHK) samples, 3 L was collected at each site (A, B, and C bottle) and the total final extract volume was 300 µL.

The mass of the sample was calculated by accounting for the mass of the bottle before and after extraction. The liter samples were extracted by using a 24-port vacuum rack with Teflon tubing attached to 6 mL SPE cartridge adapters. The 200 mg HLB SPE cartridges (Waters Corp., Milford, MA were conditioned with 8 mL methanol followed by 8 mL water. The samples were loaded onto the cartridges at ~10 mL/min, the bottle and cartridge were rinsed with 8 mL water, and air was passed through the cartridge for at least 20 min. A 24-port vacuum rack equipped with disposable polytetrafluoroethylene (PTFE) liners was used to elute the cartridges with two aliquots of 4 mL methanol into 15 mL glass conical bottom centrifuge tubes, and elution colors were noted. The samples were evaporated at 40 °C to approximately 1 mL; the extracts from the B and C bottle were washed into the A bottle centrifuge tube with two aliquots of 1 mL methanol. This combined extract was evaporated to 10 µL and then brought to a final volume of 300 µL with LCMS grade methanol. The sample was transferred to 12x32mm plastic vial with amber glass 0.1 mL insert and E-Z aluminum seal with PTFE/silicone liner lids (Wheaton, DWK Life Sciences, Millville, NJ). Extracts were held at -20 °C until overnight shipment on ice to the analytical laboratories.

Total estrogenicity of extracts was determined by using the bioluminescent yeast estrogen screen (BLYES),[5] but with minor modifications.[6] Sample extracts prepared at the OGRL were shipped on ice to the U.S. Geological Survey Eastern Ecological Science Center laboratory in Leetown, WV, and stored at -20 °C prior to the bioassay. Briefly, 20 µL of sample extract was added in triplicate to the wells of a white, solid-bottom 96-well plate and evaporated at room temperature in a Class II biological safety cabinet. Once the solvent evaporated, 200 µL of µL of a 48-hour culture of strain BLYES adjusted to 0.4 (OD_{600}) in fresh yeast minimal media (YMM leu^-, ura^-) was added to each well. A 12-point standard curve of 17β-estradiol (E2; Sigma-Aldrich CO) was included on each plate. A media control was included on all plates to establish background luminescence. Plates were covered and incubated in the dark at 30 °C for 4 hours. Luminescence was quantified by using a SpectraMax M4 microplate reader (Molecular Devices, San Jose, CA) in luminescence mode (1,000 millisecond integration time), and estrogen equivalents (E_2Eq) of each sample were determined via interpolation to a 4-parameter curve within SoftMax Pro 6.2.2 (Molecular Devices). Relative net agonistic activity per liter of sample was then calculated on the basis of sample concentration. The detection limit for this assay was 0.13 ng/L $E_2Eq_{(BLYES)}$.

Quality Assurance

Equipment blanks were prepared in the field and laboratory by processing certified organic and inorganic blank water through the sampling equipment in the same manner that field samples were collected and processed. In addition, isotope-dilution standards (IDSs) or surrogate compounds were added to all target-organic samples prior to extraction or analysis. Chemical concentrations that were outside the calibration range for each method (i.e., exceeding the analytical calibration curve or between the limit of quantitation (LOQ) and long-term method detection limit (LT-MDL),[7] were considered estimated detections and coded “E” with reduced precision and accuracy, and included as is herein. Three reported concentrations of acetophenone, eight reported concentrations of DEET, and five reported concentrations of zinc that were <10 times the field blank concentration were coded “V” to indicate that the reported concentration may have been affected by the inadvertent introduction of a small amount of the

target analyte during collection or laboratory analysis. Laboratory provided QA data for anions, cations, and trace elements were of high-quality (Table SI-1) and can be found in the associated data releases.[8]

RESULTS AND DISCUSSION

Inorganic chemicals

In general, the inorganic-chemical concentrations were dilute in all reuse waters as indicated by maximum specific conductance values in effluent (1,015 µS/cm), stormwater (425 µS/cm), and agricultural runoff (135 µS/cm; Table SI-9). Chloride (Cl^-) and bicarbonate (HCO_3^-, as alkalinity) were the most abundant anions with maximum concentrations in effluent (74.0 and 254 mg/L, respectively), stormwater (23.4 and 177 mg/L, respectively), and in agricultural runoff (2.1 and 48.6 mg/L, respectively). By comparison, Cl^- concentrations as great as 556 mg/L have been reported in previous studies of effluent,[9] urban stormwater (22.7 mg/L),[10] and agricultural runoff (17.5 mg/L).[11] No Cl^- concentrations in any of the sampled reuse waters in our study exceeded the 230 mg/L chronic aquatic life benchmark (ALB) concentration.[12] Maximum concentrations of sulfate (SO_4^{2-}) of 149 mg/L, 43.4 mg/L, and 2.1 mg/L were observed in effluent, stormwater, and agricultural runoff, respectively. Fluoride (F^-) and bromide (Br^-) concentrations in all reuse samples were either not detected or observed at low concentrations that ranged from 0.1 to 0.5 mg/L of F^- and 0.01 to 0.1 mg/L of Br^-.

Although nutrients such as phosphorous (P), nitrate, nitrite, orthophosphate, and ammonia are essential for plant development and growth, elevated levels can cause water-quality and ecological problems in aquatic systems including fish kills, toxic algal blooms, depleted oxygen, and loss of species biodiversity that are important for commerce and recreation.[13] Concentrations of total nitrogen (TN; ammonia as N + organic as N + nitrite + nitrate) and total phosphorous (P) were greatest in effluent and least in stormwater. Maximum TN concentrations were 26.1 mg/L in effluent, 13.5 mg/L in agricultural runoff, and 2.5 mg/L in stormwater. Decreasing P inputs to surface waters is a priority for federal, state, and municipal water management agencies.[14] In our study, all reuse samples of effluent, stormwater, and agricultural runoff exceeded the total P criteria concentration of 0.037 mg/L for scenic rivers in Oklahoma.[15] Total P concentrations were as large as 4.4 mg/L in effluent, 2.2 mg/L in agricultural runoff, and 0.66 mg/L in stormwater. The major cation composition of effluent and stormwater was dominated by sodium (Na^+) and calcium (Ca^{2+}), whereas agricultural runoff was dominated by Ca^{2+} and magnesium (Mg^{2+}). Maximum Na^+ concentrations were 78.6 mg/L in effluent, 26.8 mg/L in stormwater, and 0.91 mg/L in agricultural runoff (Table SI-9). Ca^{2+} concentrations were as large as 79.9 mg/L in effluent, 31.3 mg/L in stormwater, and 10.6 mg/L in agricultural runoff. Maximum Mg^{2+} concentrations were 38.4 mg/L in effluent, 23.1 mg/L in stormwater, and 5.8 mg/L in agricultural runoff.

In general, dissolved metal concentrations were relatively dilute in all reuse water samples. Maximum concentrations for arsenic (As, 5.28 ug/L) and cadmium (Cd, 0.1 ug/L) occurred in stormwater, whereas maximum concentrations for iron (Fe, 609 ug/L), nickel (Ni, 6.2 ug/L), and lead (Pb, 2.9 ug/L) occurred in agricultural runoff. Excluding the April 23, 2019,

Pb concentration of 2.9 ug/L from the I-Ag field that exceed ALB criteria (2.5 ug/L), none of the As, Cd, Fe, and Ni concentrations in any other reuse samples exceeded chronic ALB concentrations (150, 0.72, 1,000, and 52.0 ug/L, respectively).[12] However, a previous study documented that mixtures of heavy metals such as Cd, chromium (Cr), Pb, manganese (Mn), aluminum (Al), and Fe can pose human or recreational health risks even at low concentrations.[16]

ACKNOWLEDGMENTS

This research was conducted and funded by the U.S. Geological Survey (USGS) Ecosystems Mission Area and the Office of Research and Development of the U.S. Environmental Protection Agency (USEPA). The authors thank Sean Campbell, Julie Dietze, Leslie Kanagy, Clayton Raines, Nino Raynor, James Simmons, and Daniel Tush for analyses of water samples, and Darrel White and David Buchanan with the City of Chickasha for access to wastewater effluent samples. Any use of trade, product, or firm names is for descriptive purposes only and does not imply endorsement by the U.S. Government.

REFERENCES

1. Oklahoma State University [OSU] South Central Research Stations. Ag Research. 2022. https://agresearch.okstate.edu/facilities/south-central-research-station.

2. U.S. Geological Survey [USGS], 2020, USGS water data for Oklahoma, in USGS water data for the nation: U.S. Geological Survey National Water Information System database, accessed September 16, 2022, at https://doi.org/10.5066/F7P55KJN. [Information directly accessible at https://waterdata.usgs.gov/usa/nwis/uv?07328100

3. Wilde FD, Radtke DB, Gibs J, Iwatsubo RT, (Eds.). Processing of water samples (ver. 2.2). in *U.S. Geological Survey Techniques for Water Resources Investigations.* 2002. doi:10.3133/twri09A5

4. Chakravarthy VK, Babu GK, Dasu PP, Kiran GA. The role of relative response factor in related substances method development by high performance liquid chromatography (HPLC). *RASAYAN J. Chem.* 2011;4, 919-943

5. Ciparis S, Iwanowicz LR, Voshell, JR. Effects of watershed densities of animal feeding operations on nutrient concentrations and estrogenic activity in agricultural streams. *Sci. Total Environ.* 2012;414:268–276. doi:10.1016/j.scitotenv.2011.10.017

6. Sanseverino J, Gupta Rk, Layton AC, Patterson SS, Ripp SA, Saidak L, Simpson ML, Schultz TW, Sayler GS. Use of Saccharomyces cerevisiae blyes expressing bacterial bioluminescence for rapid, sensitive detection of estrogenic compounds. *Appl. Environ. Microbiol.* 2005;71:4455–4460. doi:10.1128/AEM.71.8.4455-4460.2005

7. Bonn BA. Using the U.S. Geological Survey National Water Quality Laboratory LT-

MDL to evaluate and analyze data: U.S. Geological Survey Open-File Report. 2008. doi:10.5066/P9WEPT20

8. Jaeschke JB, Campbell S, Cozzarelli IM, Gray JL, Hladik ML, Iwanowicz L, Kanagy CJ, Kanagy LK, Kolpin DW, Lane RF, McCleskey BR, Polite BF, Roth DA, Tush DL, Wilson MC, Masoner JR. Water-quality results from a wastewater reuse study: Inorganic and organic compositions of wastewater effluent and select urban and agricultural water types during rain-induced runoff, Chickasha, Oklahoma, 2018-2019. U.S. Geological Survey data release; 2022. doi:10.5066/P9WEPT20

9. Ganzenko O., Sistat P, Trellu C, Bonniol V, Rivallin M, Cretin M. Reactive electrochemical membrane for the elimination of carbamazepine in secondary effluent from wastewater treatment plant. *Chem. Eng. J.* 2021;419:129467 doi:10.1016/j.cej.2021.129467

10. Masoner JR, Kolpin DW, Cozzarelli IM, Barber LB, Burden DS, Foreman WT, Forshay KJ, Furlong ET, Groves JF, Hladik ML, Hopton ME, Jaeschke JB, Keefe SH, Krabbenhoft DP, Lowrance R, Romanok, KM, Rus DL, Selbig WR, Williams BH, Bradley PM. Urban stormwater: An overlooked pathway of extensive mixed contaminants to surface and groundwaters in the United States. *Environ Sci Technol.* 2019;53(17). doi:10.1021/acs.est.9b02867

11. Beom J, Jeung M, Choi W, Her Y, Yoon K. Characteristics of chloride loading from urban and agricultural watersheds during storm and non-storm periods. *Water Supply* 2020;21:1567-1579. doi:10.2166/ws.2020.343

12. U.S. Environmental Protection Agency. National recommended water quality criteria - aquatic life criteria table. 2020; https://www.epa.gov/wqc/national-recommended-water-quality-criteria-aquatic-life-criteria-table

13. Carpenter SR, Caraco NF, Correll DL, Howarth RW, Sharpley AN, Smith VH. Nonpoint pollution of surface waters with phosphorus and nitrogen. *Ecol. Appl.* 1998;8: 559–568. doi:10.1890/1051-0761(1998)008[0559:NPOSWW]2.0.CO;2

14. Selbig WR. Evaluation of leaf removal as a means to reduce nutrient concentrations and loads in urban stormwater. *Sci. Total Environ.* 2016;571:124–133. doi:10.1016/j.scitotenv.2016.07.003

15. U.S. Environmental Protection Agency. State Progress Toward Developing Numeric Nutrient Water Quality Criteria for Nitrogen and Phosphorus. 2021 https://www.epa.gov/sites/default/files/2014-12/documents/okwqs_chapter45.pdf

16. Ma Y, Egodawatta P, McGree J, Liu A, Goonetilleke A. Human health risk assessment of heavy metals in urban stormwater. *Sci. Total Environ.* 2016;557–558:764–772. doi:10.1016/j.scitotenv.2016.03.067

SUPPORTING INFORMATION FIGURES

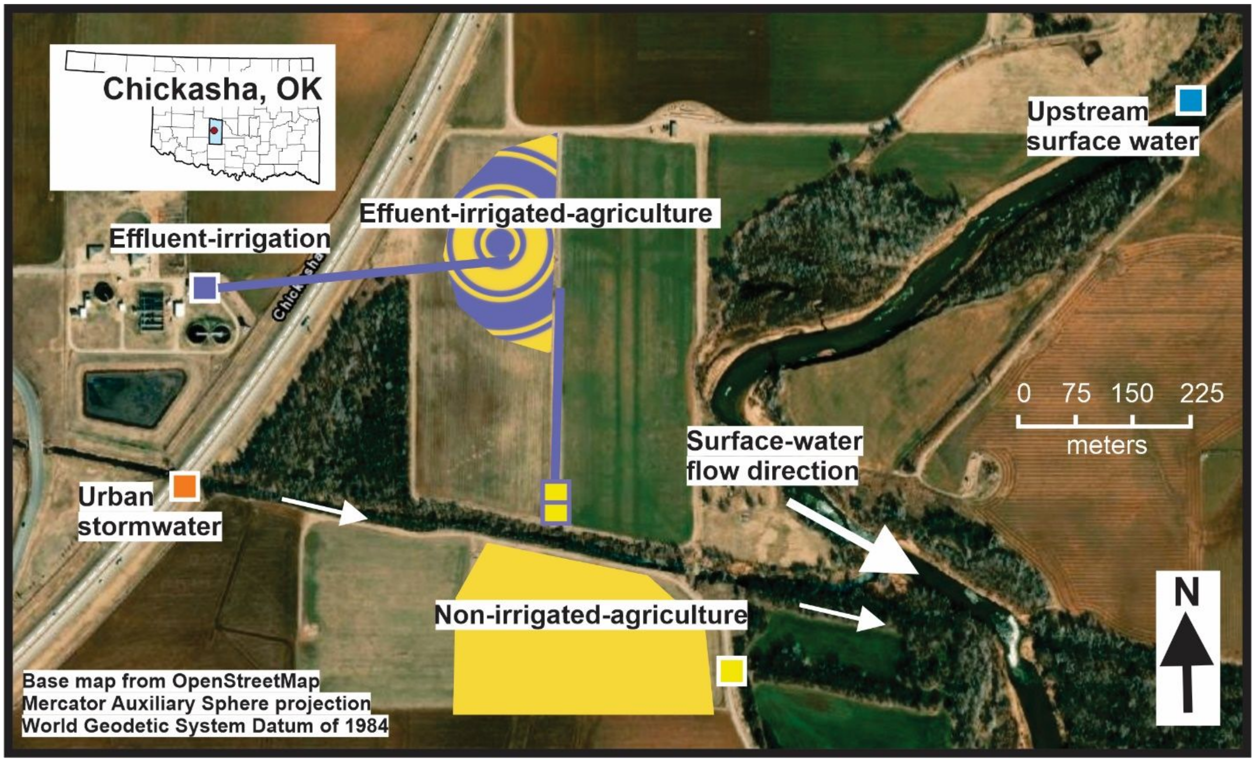

Figure SI-1. Map showing the Oklahoma State University South-Central Research Station in Chickasha, Oklahoma. Map depicts the sampling locations of source wastewater-effluent used as irrigation, urban stormwater, rain-induced agricultural runoff from effluent-irrigated (I-Ag) and non-irrigated-ag (NI-Ag) fields, and surface water upstream from all other reuse-water discharge locations.

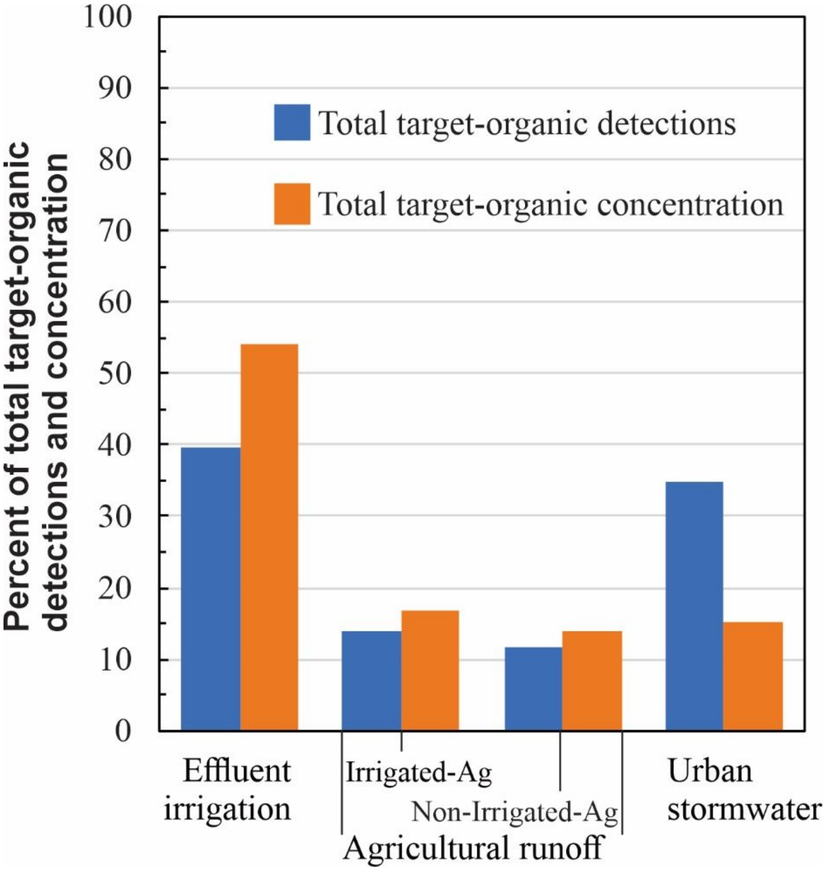

Figure SI-2. Percent of total number of detected target-organic chemicals (blue bars) and concentration (orange bars) across all effluent irrigation, agricultural (Ag) runoff, and urban stormwater samples.

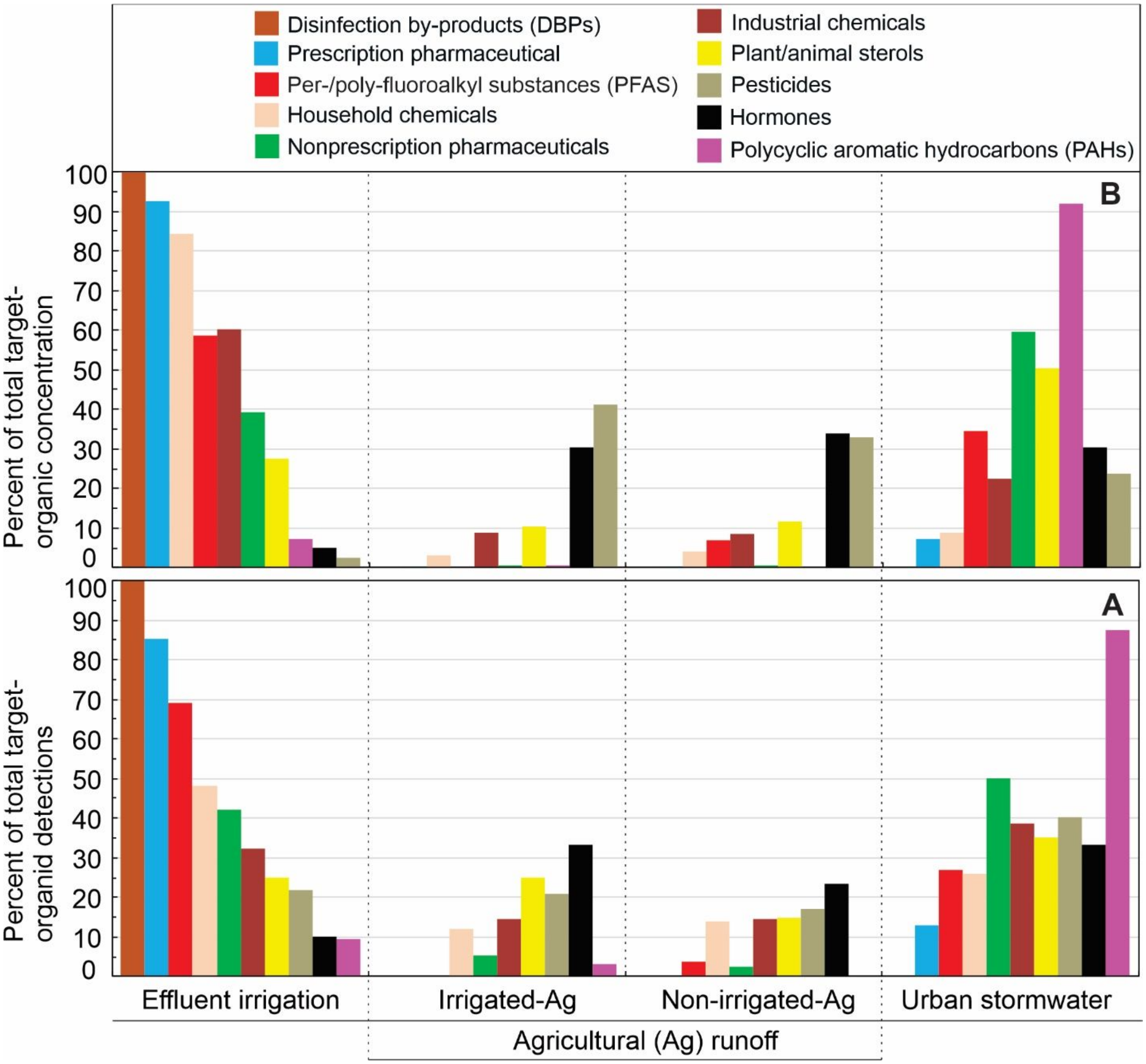

Figure SI-3. Percent of total number of target-organic detections (A) and concentration (B) across all sampled reuse waters. Grouped by chemical class and sorted from left to right by decreasing total number of detections in effluent irrigation.

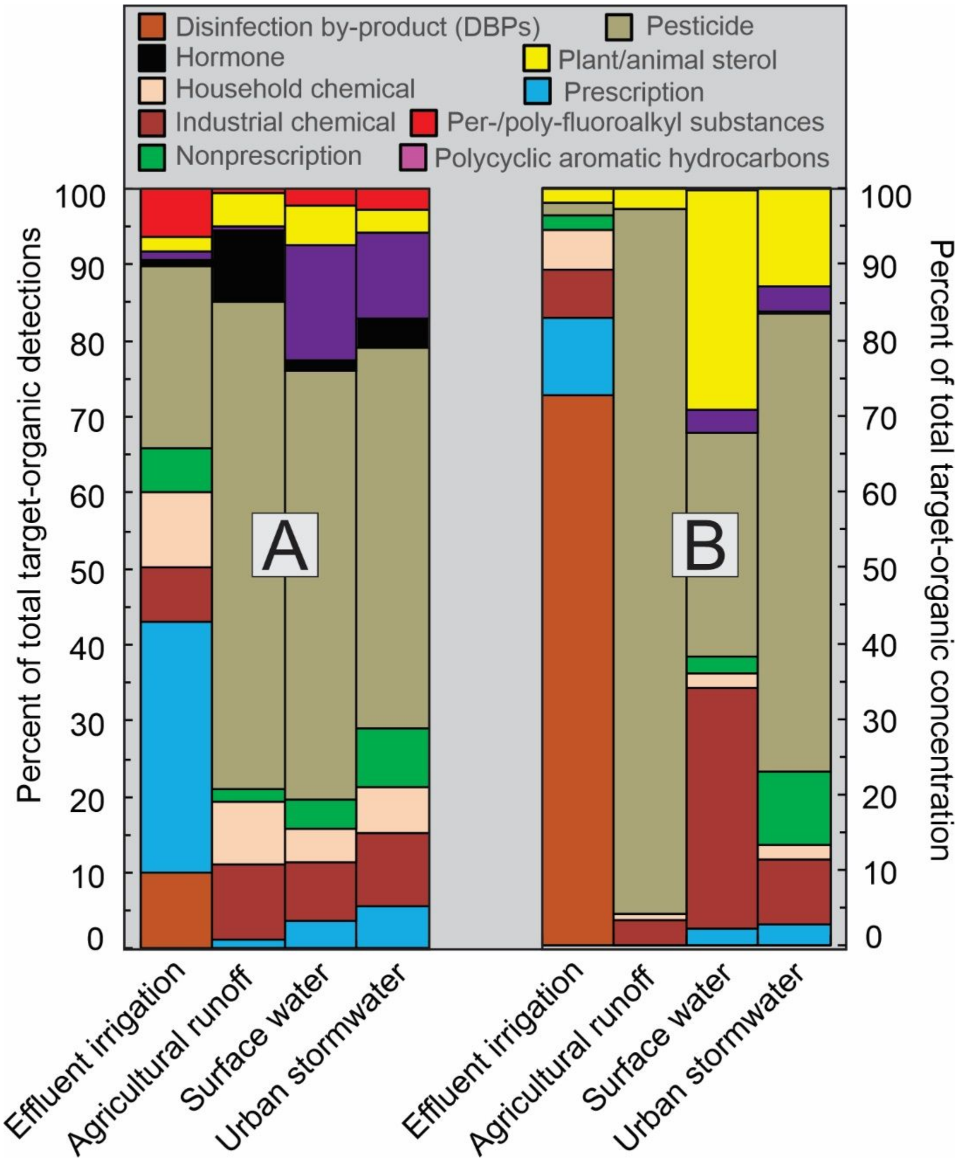

Figure SI-4. Percent of total target-organic detections (A) and concentration (B) for individual sampled sites of effluent irrigation, urban stormwater, agricultural runoff, and surface water.

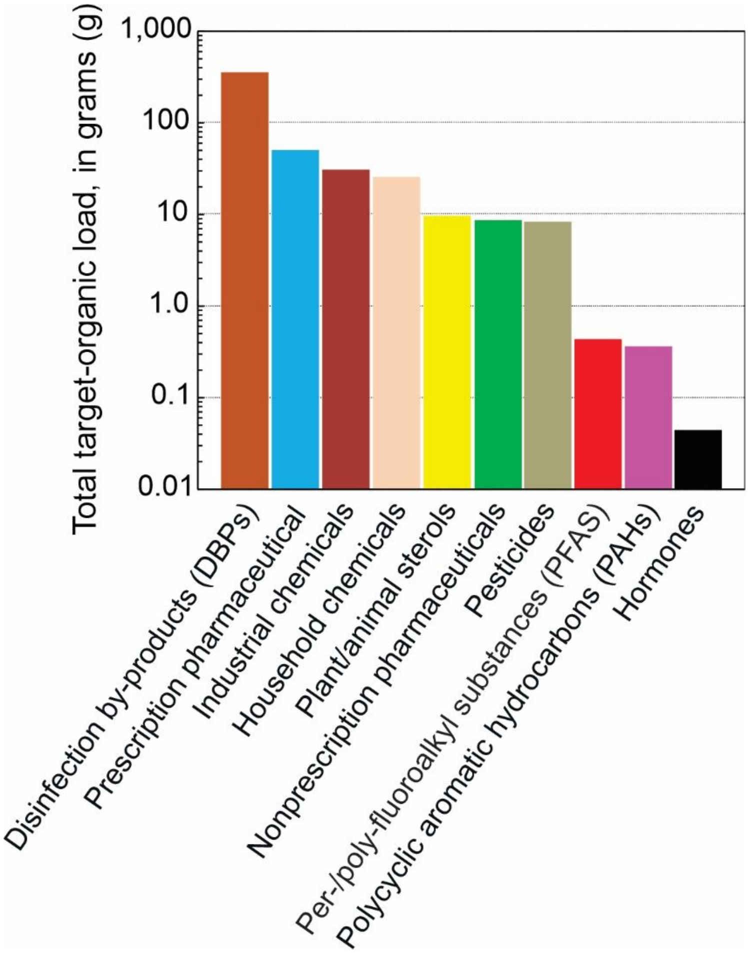

Figure SI-5. Total target-organic wastewater effluent-irrigation load (491 g) applied to irrigated-ag (I-Ag) field over a 24-day period from July 15 to August 8, 2019, grouped by chemical class and sorted from left to right by decreasing load amount.

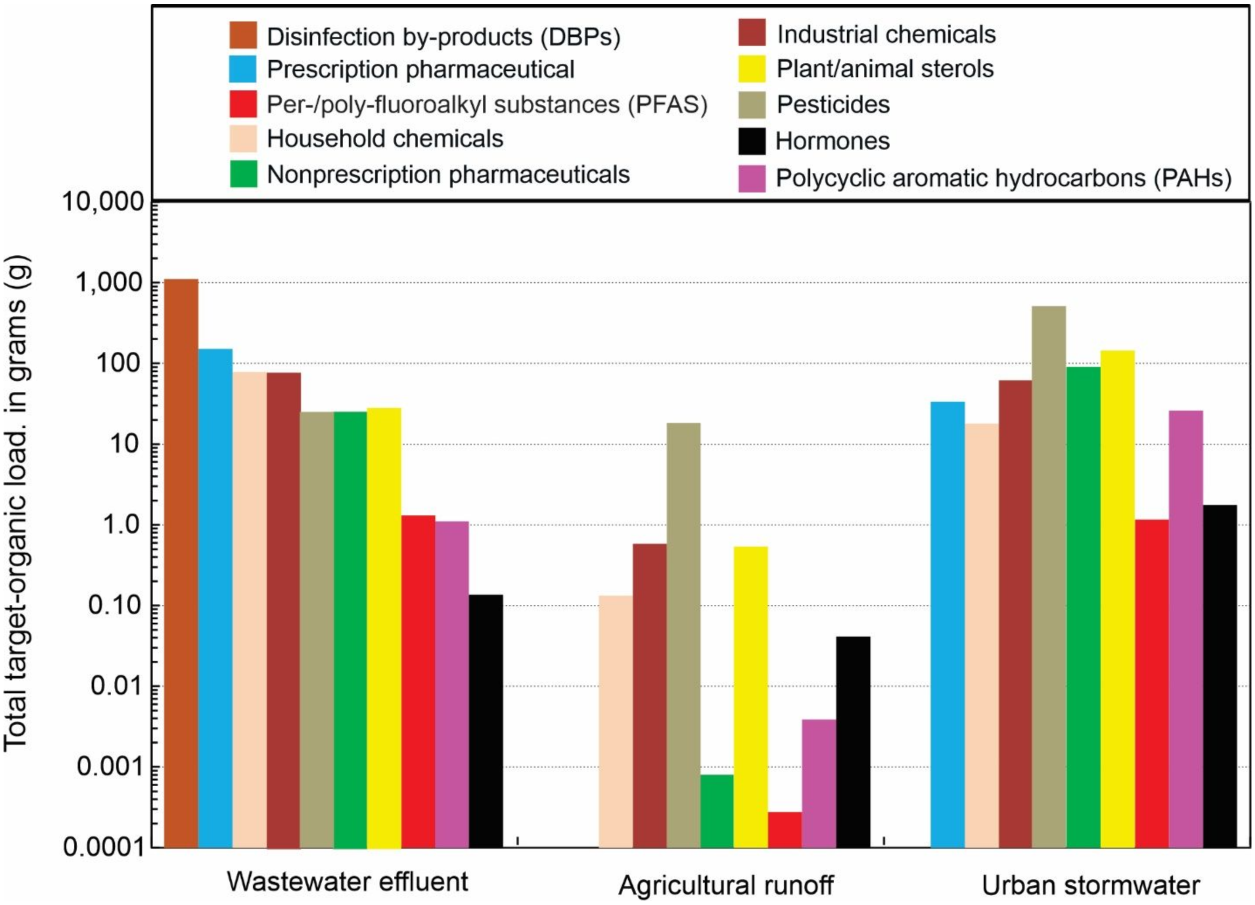

Figure SI-6. Total target-organic load contributions to receiving surface water from wastewater effluent, urban stormwater, and agricultural runoff from sampled sites, grouped by chemical class and sorted from left to right by decreasing load amounts from wastewater-effluent irrigation.

SUPPORTING INFORMATION TABLES

Supporting information tables are contained in the associated supporting information spreadsheet “SI-Tables.xlsx”. The individual table titles are:

Table SI-1. Site and hydrologic information for samples of wastewater-effluent irrigation, urban stormwater, agricultural field runoff, and whole corn plant samples (above ground). Sorted by field name and then by date.

Table SI-2. Laboratory quality-control measurements from organic analyses of reagent-blank samples typically analyzed once every ten field samples. Sorted alphabetically by analytical method then by parameter name.

Table SI-3. Laboratory quality-control recoveries (in percent) from organic analyses of reagent-spike samples typically analyzed once every ten field samples. Sorted alphabetically by analytical method then by parameter name.

Table SI-4. Field quality-control measurements from inorganic and organic analyses of two equipment blank samples. Sorted alphabetically by analytical method then by parameter name.

Table SI-5. Organic chemicals summary statistics of recoveries for isotope-dilution standards (IDSs), surrogate compounds, and method reagent-spikes added to field and quality-assurance samples.

Table SI-6. Concentrations of detected target-organic chemicals and nonvolatile dissolved organic carbon measured in samples of wastewater-effluent irrigation, stormwater runoff from agricultural fields and urban landscape. Sorted alphabetically by analytical method then by parameter name.

Table SI-7. Target-organic chemicals not detected in samples of wastewater-effluent irrigation, stormwater runoff from agricultural fields and urban landscape. Sorted alphabetically by analytical method then by parameter name.

Table SI-8. Total number of detections, concentrations, and loads for organic-chemical classes.

Table SI-9. Water properties and measured concentrations of anions, cations, nutrients, trace metals, and rare-earth elements in samples of wastewater-effluent irrigation and stormwater runoff from agricultural fields and urban landscape. Sorted alphabetically by analytical method then by parameter name.

Table SI-10. Pharmaceuticals and per-/poly-fluoroalkyl substances (PFAS) concentrations and percent recoveries for isotope-dilution/surrogate standards analyzed by SGS AXYS Analytical Services in whole corn plant (above ground) samples collected at harvest for the 2019 growing season. Sorted alphabetically by analytical method then by parameter name.
